# The value of ^99m^Tc-methylene diphosphonate single photon emission computed tomography/computed tomography in diagnosis of fibrous dysplasia

**DOI:** 10.1186/s12880-017-0218-4

**Published:** 2017-07-24

**Authors:** Linqi Zhang, Qiao He, Wei Li, Rusen Zhang

**Affiliations:** 1 Department of Nuclear Medicine, Affiliated Cancer Hospital&Institute of Guangzhou Medical University, 78 Hengzhigang Road, Guangzhou, 510095 Guangdong province People’s Republic of China; 2 Department of Nuclear Medicine, the First Affiliated Hospital of Sun Yat-Sen University, 58 Zhongshan Er Road, Guangzhou, 510080 Guangdong province People’s Republic of China

**Keywords:** Fibrous dysplasia, ^99m^Tc-MDP, Single photon emission computed tomography, Computed tomography

## Abstract

**Background:**

Fibrous dysplasia (FD) is a rare benign bone disorder in which the normal bone is replaced by immature fibro-osseous tissue. However, some case reports have reported that FD showed significantly increased ^99m^Tc-methylene diphosphonate (^99m^Tc-MDP) uptake on whole-body bone scintigraphy (WBS), which may mimic bone metastasis or skeletal involvement of the patients with known cancer. Thus, the purpose of present study is to observe the reliable characteristics and usefulness of single photon emission computed tomography/computed tomography (SPECT/CT) for the diagnosis of FD.

**Methods:**

This was a retrospective review of 21 patients with FD (14 males and 7 females, mean age 51.2 ± 12.5 years) who were referred to have WBS to determine whether there was any osseous metastasis. WBS and SPECT/CT images were independently interpreted by two experienced nuclear medicine physician together with a diagnostic radiologist. In cases of discrepancy, consensus was obtained by a joint reading. The final diagnosis was based on biopsy proof and radiologic follow-up over at least 1 year.

**Results:**

The lesions of FD were most frequently found in craniofacial region (15/21). Eighteen of the 21 (85.7%) cases showed moderate and high metabolism on WBS (compared to sternum). On CT imaging, GGO and expansion were the most common finding, were noted in 90.5% and 85.7% of the patients. Lytic lesions were present in 61.9% of the patients, and sclerosis was present in 38.1% of the patients. Cortical disruption was not seen in any patient.

**Conclusions:**

FD has certain characteristic appearance on SPECT/CT. It should be enrolled in the differential diagnoses when lesions show elevated ^99m^Tc-MDP uptake on WBS. For SPECT/CT, the CT features of GGO and expansion in the areas of abnormal radiotracer uptake are helpful for the diagnosis of FD.

## Background

Fibrous dysplasia (FD) is a rare benign bone disorder in which the normal bone is replaced by immature fibro-osseous tissue. The actual prevalence of FD is difficult to estimate, but it may affect about 1/30,000 persons with a similar distribution around the world. The disease may involve single bone (monostotic FD, 70%) or multiple bones (polyostotic FD, 30%) with a predilection for the craniofacial bones and ribs. Patients are usually asymptomatic and detected incidentally on imaging studies that are performed for other purposes. In rare symptomatic cases, FD can present as bone pain, deformity, or pathologic fracture [1, 2]. ^99m^Tc-MDP (^99m^Tc-labeled methylene diphosphonate) whole-body bone scans (WBS) has been widely used for detection of metastasis for various malignant diseases. However, some case reports have reported that FD showed significantly increased ^99m^Tc-MDP uptake, which may mimic bone metastasis or skeletal involvement of the patients with known cancer [3–5]. Therefore, active diagnosis and radiological familiarity of FD are thought to be essential for distinguishing bone metastasis and preventing unnecessary examinations or therapy. Single photon emission computed tomography/computed tomography (SPECT/CT) offers the opportunity to obtain diagnostic-quality CT and SPECT images, hence enabling more accurate localization and characterization of SPECT lesions using the CT component. However, the SPECT/CT features of FD have not been summarized. In present study, we wished to observe the reliable characteristics and usefulness of SPECT/CT in a larger cohort of patients with FD.

## Methods

### Patients

A total of 27,859 patients underwent ^99m^Tc-MDP WBS from March 2009 to January 2017 at Department of Nuclear Medicine. Among which, there were 8517 patients had SPECT/CT for further evaluation. Of these patients, twenty one patients (fourteen males and seven females, mean age 51.2 ± 12.5 years, age range 23 ~ 70 years) found to have FD were recruited in the study. In 13 cases, the clinician performed biopsies to determine whether there was any osseous metastasis, because the anatomic site of the lesion was easily accessible. Pathologic analysis confirmed the diagnosis of FD. In 8 cases, the patients had been diagnosed based on radiologic investigations (SPECT/CT and/or MRI) and follow up at least one year.

### SPETCT/CT scanning

All examinations were carried out using a SPECT/CT scanner (Philips, Netherlands,16-slice diagnostic CT). The whole-body scan was performed 3 h after intravenous injection of 15 ~ 25 mCi ^99m^Tc-MDP. The images were immediately reviewed by a nuclear medicine radiologist after image acquisition. If areas of abnormal radiotracer uptake were detected, the patient then proceeded directly for SPECT/CT for anatomic location and attenuation correction of the areas. The acquisition parameters for CT were as following: 140KeV, window width 15%, pitch 1.25, and slice thickness 5.0 mm. Directly after CT imaging, the SPECT acquisition protocol was started. The SPECT/CT imaging was integrated and analyzed by using Philips Jet Steam Workspace integrated program. The coronal, sagittal and transverse plane of SPECT, CT and SPECT/CT was evaluated, respectively.

### Imaging analysis

The WBS and SPECT/CT images were independently interpreted by two experienced nuclear medicine physician together with a diagnostic radiologist. In cases of discrepancy, consensus was obtained by a joint reading. It was considered high metabolism if the lesion showing uptake of ^99m^Tc-MDP higher than that of sternum on WBS images, equal to that of sternum was considered moderate metabolism, and lower than that of sternum was considered low metabolism. The following radiologic features were evaluated on CT images: ground-glass opacity (GGO), expansion, lytic lesions, sclerosis, and cortical disruption (presence or absence).

### Statistical analysis

Categorical data are expressed as numbers and frequency (%). Continuous data are expressed as means and standard deviations. All the statistical tests were performed using SPSS Statistics 17.0 (SPSS Inc., Chicago, IL, USA) software.

## Result

### Patient population

A summary of clinical characteristics (including age, gender, known malignancy and diagnostic method), WBS and SPECT/CT findings (including location and CT features) of all 21 patients with FD were given in Table 1. Nineteen of 21 patients (90.5%) were asymptomatic and detected incidentally on WBS. The remaining 2 patients (9.5%) presented with aspecific symptoms: one (patient 5) with nasal obstruction, and another (patient 12) with dull pain in left tibia. Only one patient (patient 14) (4.8%) was polyostotic and other 20 patients (95.2%) were monostotic. Lesions were most frequently found in craniofacial region, accounting for 71.4% (15/21) of patients, five of the patients in the skull, three in the maxillary, three in the mandible, three in sphenoid, and one patient showed conjoint sphenoid and ethmoid involvement. The remaining 6 patients, one patient with polyostotic lesion involvement of rib and vertebra, other 5 patients with solitary lesion in rib (*n* = 3), ischium (*n* = 1), and long bone (*n* = 1).

**Table 1:** Clinical data and SPECT/CT findings of FD in 21 patients with known cancer

Pat. No.	Localization	Known cancer	Diagnostic Method	GGO	Expansion	Lytic	Sclerosis	Cortical Disruption
1	Mandible	Lung cancer	Biopsy	+	+	−	−	−
2	Sphenoid	Gastric lymphoma	radiologic follow-up	+	+	−	−	−
3	Maxillary	Lung cancer	Biopsy	+	+	+	−	−
4	Sphenoid	HCC	radiologic follow-up	+	+	+	−	−
5	conjoint sphenoid and ethmoid	HCC	Biopsy	+	+	−	+	−
6	L. Rib	NPC	Biopsy	−	+	+	+	−
7	L. Frontal bone	Lung cancer	Biopsy	+	+	+	−	−
8	R. Parietal bone	ESCC	Biopsy	+	+	−	+	−
9	R. Frontal bone	LSCC	Biopsy	+	+	+	−	−
10	R. Ischium	Breast cancer	Biopsy	−	−	+	−	−
11	R. Occipital bone	NPC	Biopsy	+	−	+	−	−
12	L. Tibia	NPC	Biopsy	+	−	−	+	−
13	Maxillary	ESCC	radiologic follow-up	+	+	−	+	−
14	Rib, vertebra	HCC	radiologic follow-up	+	+	+	−	−
15	R. Frontal bone	ESCC	Biopsy	+	+	+	−	−
16	R. Rib	NPC	radiologic follow-up	+	+	+	−	−
17	Maxillary	Lung cancer	Biopsy	+	+	+	+	−
18	Maxillary	NPC	radiologic follow-up	+	+	−	−	−
19	Mandible	Cervical cancer	radiologic follow-up	+	+	−	−	−
20	Sphenoid	LSCC	radiologic follow-up	+	+	+	+	−
21	R. Rib	ESCC	Biopsy	+	+	+	+	−

*Pat. No* patient number, *R* right, *L* left, *HCC* hepatocellular carcinoma, *NPC* nasopharyngeal carcinoma, *ESCC* esophageal squamous carcinoma, *LSCC* laryngeal squamous carcinoma, *GGO* ground-glass opacity, *+* positive, *−* negative.

### WBS and SPECT/CT findings

**Fig. 1:**
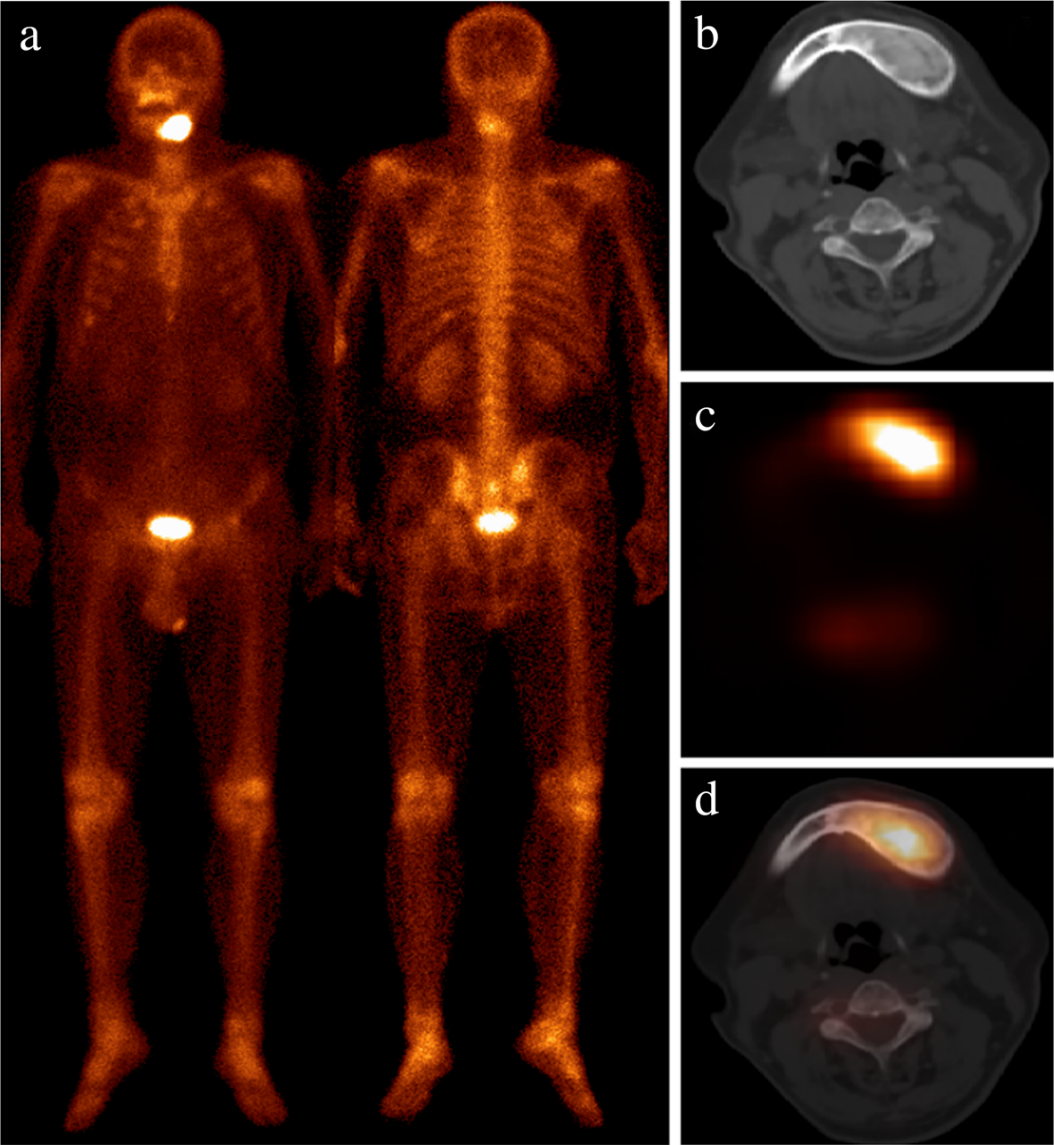
Patient 1 presented with newly diagnosed lung cancer. The WBS (**a**) demonstrated markedly increasing ^99m^Tc-MDP uptake in the left facial bone. Axial CT (**b**), SPECT (**c**), and hybrid SPECT/spiral CT imaging (**d**) demonstrated increasing ^99m^Tc-MDP uptake corresponding to expansion and ground glass density on mandible. A biopsy was subsequently performed and pathologic analysis confirmed the diagnosis of FD

**Fig. 2:**
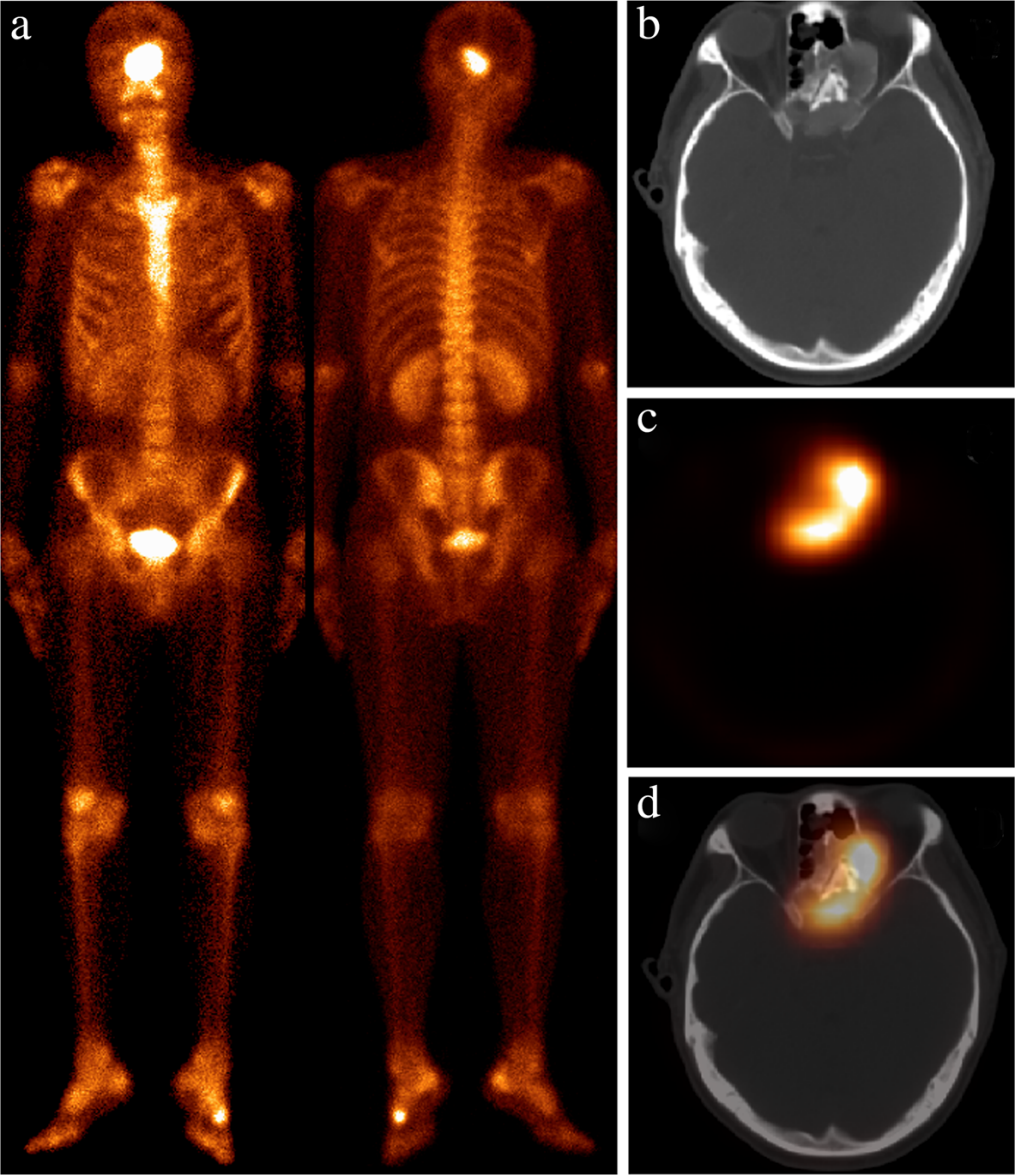
Patient 5 presented with HCC. The WBS (**a**) demonstrated markedly increasing ^99m^Tc-MDP uptake in the base of skull. Axial CT (**b**), SPECT (**c**), and hybrid SPECT/spiral CT imaging (**d**) showed an expansile lesion, which presented with GGO and ill-defined borders in the conjoint sphenoid and ethmoid. The clinician decided immediately to perform a biopsy, because the anatomic site of the lesion was easily accessible by endoscopic sinus. Pathologic analysis confirmed the diagnosis of FD

**Fig. 3:**
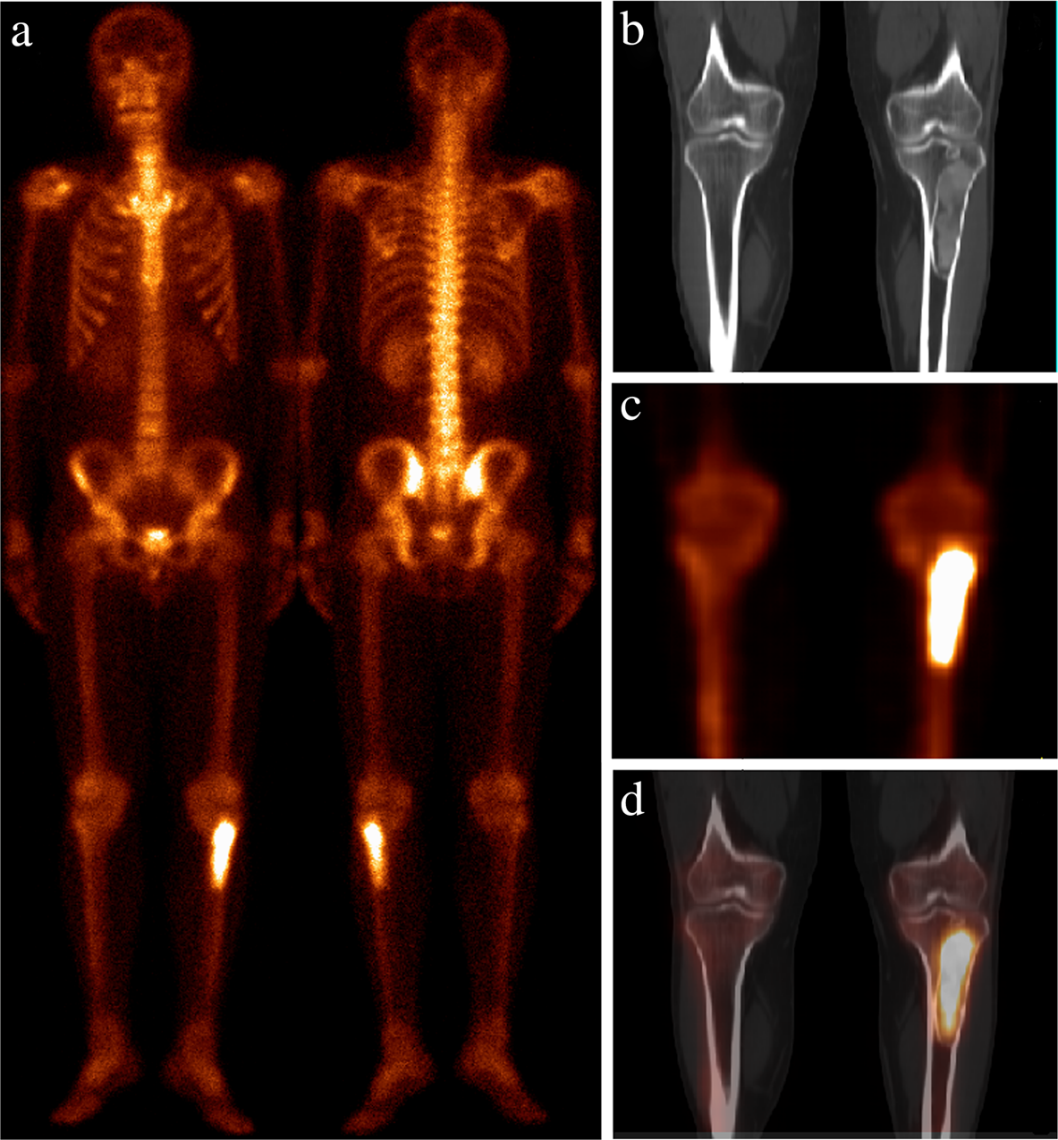
Patient 12 presented with NPC. On the WBS (**a**), there is an area of abnormal ^99m^Tc-MDP uptake seen in the left upper tibia, which may mimic bone metastasis. No additional area of abnormal ^99m^Tc-MDP uptake was identified on the remainder of the skeleton. Axial CT (**b**), SPECT (**c**), and hybrid SPECT/spiral CT imaging (**d**) depicted show increasing ^99m^Tc-MDP uptake corresponding to a sclerotic lesion with GGO localized in the left tibia. Based on the imaging finding which was suspicious for primary bone malignancy. A biopsy was subsequently performed and pathologic analysis confirmed the diagnosis of FD

**Fig. 4:**
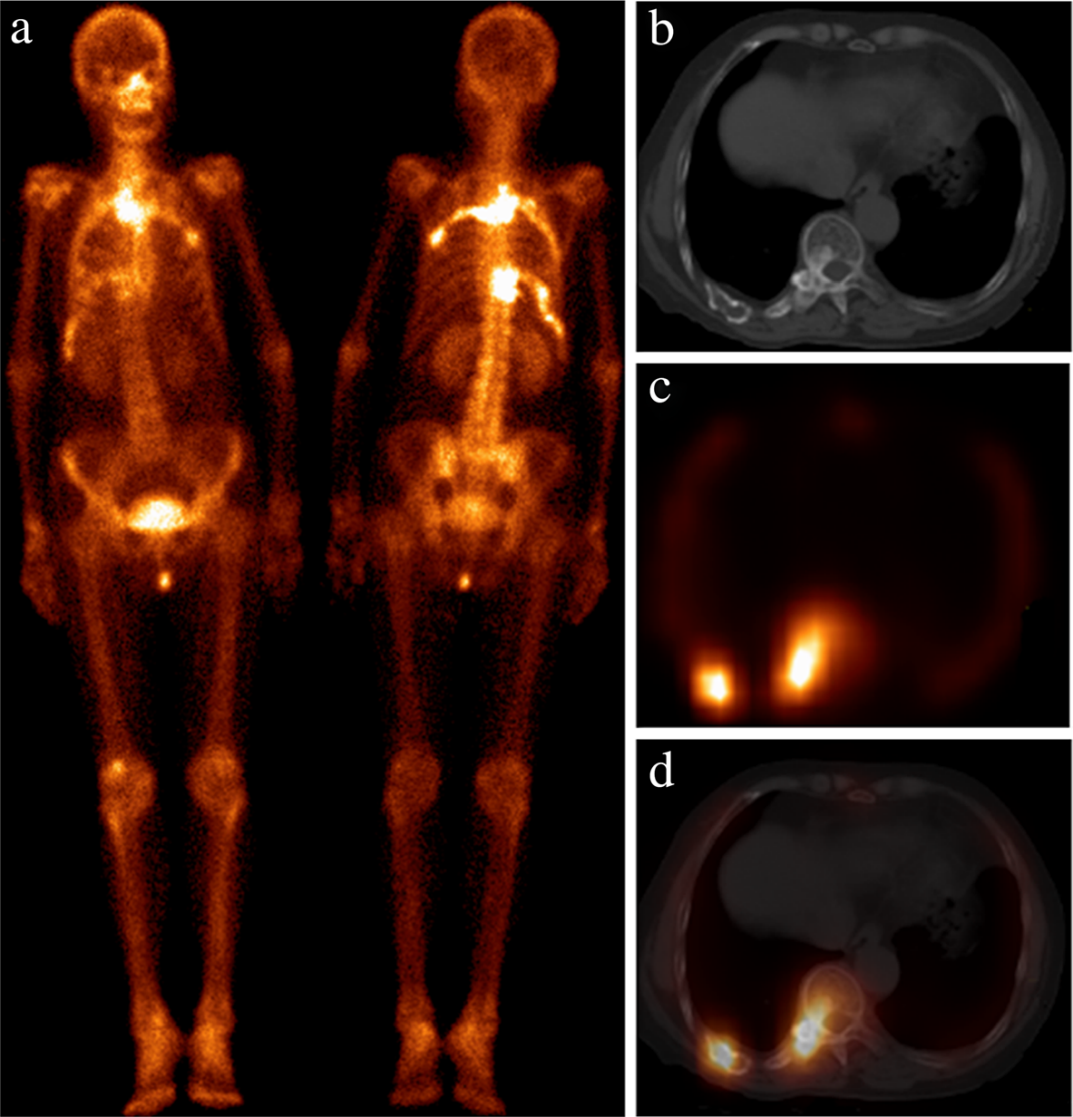
Patient 14 presented with HCC. On the WBS (**a**), there were multiple areas of abnormal ^99m^Tc-MDP uptake seen in the bilateral ribs and thoracic vertebra, which may mimic multiple bone metastasise. Axial CT (**b**), SPECT (**c**), and hybrid SPECT/spiral CT imaging (**d**) depicted increasing ^99m^Tc-MDP uptake corresponding to an expansile and lytic lesion with GGO in the lesion of bilateral ribs and thoracic vertebra. During the 2-year follow-up, no difference was detected in the WBS and CT image. The diagnosis of FD was established by a combined assessment of clinical and radiologic follow-up

Summary SPECT/CT features of 21 patients with FD were shown in Table 2. On WBS, all the lesions showed increased uptake of ^99m^Tc-MDP. Among which, there were 18 of the 21 (85.7%) cases showed moderate and high metabolism (compared to sternum). Both GGO and Expansion were noted in vast majority of patients. GGO was present in 90.5% of patients (19/21, Figs. 1, 2, 3 and 4). Expansion was present in 85.7% of patients (18/21, Figs. 1, 2, 4). Lytic lesions were present in 13 patients (13/21, 61.9%, Fig. 4) with FD. Sclerosis was noted in only 8 patients (38.1%, Figs. 2, 3) with FD. Cortical disruption was not seen in any patients.

**Table 2:** Summary SPECT/CT features of 21 patients with FD

**SPECT/CT f**indings	No. Patients(n)	Percentage (%)
Moderate and high metabolism	18	85.7%
GGO	19	90.5%
Expansion	18	85.7%
Lytic	13	61.9%
Sclerosis	8	38.1%
Cortical disruption	0	0

*GGO* ground-glass opacity

## Discussion

WBS using ^99m^Tc-MDP is one of the most frequently performed radionuclide procedures. Its excellent sensitivity makes it useful in screening for generalized bone abnormalities, but with lower specificity due to trauma, inflammation, and other malignant or benign bone diseases [6–8]. In some previous case reports, it has been recognized as being metabolically active on WBS [3–5]. However, the diagnosis of FD could not always be established only by WBS, which often needs to combine with an anatomical imaging (such as X-ray, CT, or MRI). Hybrid SPECT/spiral CT offers the opportunity to obtain diagnostic-quality CT and SPECT images, which provides a clear view of the anatomic sites of the lesions showed elevated ^99m^Tc-MDP uptake [9, 10].

Of the cases examined in present study, all the patients showed increased uptake of ^99m^Tc-MDP on WBS. Eighteen of the 21 (85.7%) cases showed moderate and high metabolism. The mechanism of different degree of ^99m^Tc-MDP metabolism of FD is unclear. One reason can be accounted for that. As we known, FD is a developmental failure in the remodeling of primitive bone to mature lamellar bone. Fibroblasts are the predominant proliferating cells in FD lesions, and the different degree of ^99m^Tc-MDP metabolism among FD may be due to the difference in the amount of proliferating fibroblasts or their metabolic turnover [11].Tracers uptake of FD have also been found in PET/CT, including radionuclide of ^68^Ga, ^18^F–fluorodeoxyglucose and ^11^C–choline [12–14].

On SPECT/CT imaging, GGO and expansion were the most common findings, noted in 90.5% and 85.7% of the cases. Lytic lesions were present in 61.9% of the cases, and sclerosis was present in 38.1% of the cases. Cortical disruption was not seen in any patients. Some previous studies have reported that the typical CT features of FD are ground-glass opacity (GGO) and expansion of the bone, due to the simultaneous presence of bone trabeculae and fibrous tissue [15–17]. Given these result, GGO and expansion appear to be reliable CT feature for diagnosis of fibro-osseous lesions. The differential diagnosis should include the other fibro-osseous diseases (ossifying fibroma and osseous dysplasia) and Paget disease [18].

The management of FD is not surgical unless it causes progressive deformity, cranial nerve compromise, pain, or malignant transformation. A malignant transformation of FD is rare, which occurs less than 1% of cases [19, 20]. In present study, the clinicians performed biopsy or surgery for 13 of the patients. All pathological results were reported as fibrous dysplasia, and no malignancy changes were detected. Some previous studies have reported that a history of radiotherapy may result in malignant transformation of FD [21]. Long-term medical imaging monitoring of FD is essential, especially in patient with a history of radiotherapy.

## Conclusions

In conclusion, FD has certain characteristic appearance on SPECT/CT. It should be enrolled in the differential diagnoses when lesions show elevated ^99m^Tc-MDP uptake on WBS image. On SPECT/CT image, the CT features of GGO and expansion in the areas of abnormal radiotracer uptake are helpful for the diagnosis of FD.

## Abbreviations

^99m^Tc-MDP: ^99m^Tc-methylene diphosphonate
FD: Fibrous dysplasia
SPECT/CT: Single photon emission computed tomography/computed tomography.
WBS: whole-body bone scintigraphy
